# Managing Malignant Pleural Mesothelioma in the Age of Personalized Medicine: Where Are We and What Is Still Missing?

**DOI:** 10.3390/cancers14225540

**Published:** 2022-11-11

**Authors:** Giulia Maria Stella, Chandra Bortolotto

**Affiliations:** 1 Department of Internal Medicine and Medical Therapeutics, University of Pavia Medical School, 27100 Pavia, Italy; 2 Cardio-Thoraco-Vascular Department, Unit of Respiratory Diseases, IRCCS Policlinico San Matteo Foundation and University of Pavia Medical School, 27100 Pavia, Italy; 3 Department of Clinical, Surgical, Diagnostic and Pediatric Sciences, University of Pavia, 27100 Pavia, Italy; 4 Department of Diagnostic and Imaging Services, Unit of Radiology, and University of Pavia Medical School, 27100 Pavia, Italy

Malignant Mesothelioma (MM) is an aggressive neoplasm of the pleural mesothelium, less frequently peritoneal and exceptionally of the vaginal tunic of the testicle and pericardium. Asbestos is the main etiopathogenetic agent, and occupational exposure can be documented in 70–80% of affected patients. Overall, malignant pleural mesothelioma (MPM) is a rare neoplasm with a poor prognosis. In European countries, the incidence of MPM is around 1.1–1.25 new cases per 100,000 inhabitants. It is important to underline that it constitutes a pathology that is currently orphan both in terms of pathogenic mechanisms and effective therapies. Malignant Pleural Mesothelioma (MPM) is classified by the European Union (EU) as a rare disease, and it is included in the European Reference Network (ERN) for the lung, dedicated to rare respiratory diseases (see website for ERN-LUNG/Rare respiratory diseases). Unfortunately, the disease is of reduced interest to the pharmaceutical industry due to the low global incidence and to the fact that the use of asbestos is now banned, and consequently, its impact as a cause of MPM is expected to be lower in the near future. However, at present, the incidence of this pathology is highly variable based on the geographical distribution which follows the previous activity of industrial plants dedicated to the processing of asbestos-cement fibers.

Personalized medicine and molecular oncology approaches have now reached and impacted the management of MPM. The twofold scope of the Special Issue dedicated to MPM is, on one hand, to highlight the critical issues which deserve deeper discussion and, on the other, to focus on the novel and most promising diagnostic and therapeutic approaches.

The first point regards the preclinical models used to develop novel therapeutic strategies. MPM has a predominantly local pattern of progression, and the disease rarely progresses with distant dissemination. It is this intrinsic feature of the disease that supports the rationale for local (intrapleural) administration of drugs. The mouse model generally represents the most frequently used animal model for experimentation in MPM. However, small animals, such as mice, do not always represent a reliable model. For instance, intrapleural treatment is difficult from a surgical point of view, both for the execution of an adequate xenotransplant of human mesothelioma in the pleura, and for the execution of subsequent loco-regional treatment. It is important to underline how the use of this species is relevant in the first place for targeting, gene manipulation and gene expression profiling studies, given the high homology of the mouse genome with the human one and how the data obtained on these models have allowed many elucidations regarding the biomolecular and pathogenetic mechanisms of the disease. However, a mouse is not a “little human”, and it appears evident that the results obtained from such a model cannot simply be extrapolated to the human condition, mainly if related to peritumoral stroma, and that the animal model to be used must be chosen appropriately from time to time based on the specific research objective. In this line, the work conducted by Affatato R. and collaborators reported the generation of a patient-derived preclinical xenograft (PDX) model representative of the different MPM histotypes. Interestingly, the mice were poorly responsive to cisplatin. From one of the PDXs, 2D cultures and 3D organoids were derived, and they were able to retain phenotypical features of the correspondent PDX and the human primary tumor [1]. Overall, these preclinical models offer a powerful tool suitable for the discovery of new treatments and to screen combinatorial approaches.

Secondly, the identification of actionable targets against MPM is still difficult and—in some aspects—obscure. Asbestos is essentially a non-mutagenic carcinogen, and till now, three different strategies have been developed, with suboptimal results. Most of the genetic alterations in MPM are attributable to a loss of function of tumor suppressor genes or caretaker genes (such as BAP1 and NF2 CDKN2A). However, in the absence of a context of oncogenic addiction, pharmaceutical targeting of surrogate activated transducers (mTor inhibitors, FAK inhibitors) might hold the key to improving MPM therapy. The article by H. Yang and coll. focused on the loss-of-function mutations in negative regulators of the Hippo-YAP pathway, such as NF2 and LATS1/2, which are known to promote MPM. The authors found that the loss of function in NF2 is linked to the aberrant activation of Hippo-YAP-independent signaling. These features defined distinct MPM subsets with specific prognostic and therapeutic implications [2]. Preclinical findings also suggest a potential role of epigenetic alteration in MPM onset and progression. Another option is to block soluble factors that are known to promote MPM growth or their cellular receptors, such as VEGFR and its inhibitor bevacizumab. The third and most recent strategy aims at targeting tumor-associated surface antigens and stimulating the immune/inflammatory system to autonomously eliminate MPM cells, with most interest now focused on immune checkpoint inhibition. The latter is extensively treated in the review by R. Terenziani et al. The authors described the most promising emerging immunotherapy treatments for MPM, from ICIs to engineered T cells to express chimeric antigen receptors (CARs) and dendritic cells (DCs) vaccines, with the main focus on both the preclinical basis and clinical exploitation. Overall, asbestos is a non-mutagenic carcinogen, and the unique MPM tumor microenvironment is a major player in tumor progression, the study of which deserves major interest from pathogenic and pharmacological perspectives. The research conducted by R. Vidergar and collaborators shone new light on the role of hyaluronic acid (HA). They showed that MPM primary cells isolated from patients can synthesize HA by themselves. In addition, complement protein C1q bound to HA could impinge upon HA homeostasis by upregulating the synthetase HAS3 both at the mRNA and the protein levels, while coherently high expression of HAS3 correlated with poorer MPM outcomes [3]. Overall, the findings of this work confirmed that C1q bound to HA may exert pro-tumorigenic activity and identified HAS3 as novel powerful target in MPM. The observation that the tumor mutational burden (TMB) is extremely low in MPM sustains the rationale of the opinion paper presented by H. Brossel et al. The authors reasoned that the stimulation of mechanisms of the DNA damage tolerance (DDT) pathways could increase the frequency of non-synonymous mutations that are, in turn, processed and presented by the MHC-I complex. Thus, they proposed to exploit a transient activation of the error-prone DDT to generate neoantigens while preserving a fully competent antitumor immune response [4]. Another option to efficiency target MPM is discussed in the work published by Johnson B. et al., which shows that the combination of small molecule inhibitor-targeting agents of survivin and FAK with tumor-suppressor miRNA mimics might constitute promising efficacy as a potential combinational treatment [5].

Some relevant questions remain to be clarified. The first issue regards the development of efficient strategies for the early diagnosis of MPM. This point raises the need, on one hand, to seriously promote transcriptome profile analysis to identify prognostic profiles and, on the other, for a deeper investigation and definition of the MPM-initiating cells. Overall, these data should then be integrated with those regarding genetic susceptibility risk, in order to design and promote effective screening programs among asbestos-exposed populations.

Computed tomography (CT) is currently used as the first imaging modality for pleural lesion diagnosis, staging, and monitoring after therapies, but it has some limits in loco-regional evaluation, and it is affected by a considerable interobserver variability. In particular, CT has a low soft-tissue contrast, and this may result in the determination of an inaccurate local extent or an imprecise pleural and adjacent involvement; all efforts to make an early diagnosis in exposed populations has failed so far. Chest magnetic resonance (MR) may be the solution to overcome some limits of CT, as in recent years, MR has been demonstrated to have a higher spatial resolution and a better soft-tissue contrast than CT, with an increase in sensitivity in depicting diaphragm and chest-wall invasion. The lack of an efficient strategy for the early diagnosis of MPM might be overcome by the superior ability of MR in depicting, with both morphological and functional evaluations, the early signs of the disease. Radiomics and artificial intelligence could be potential tools to assist in the interpretation of these complex findings, providing objective and reproducible quantitative information both from CT and MR. It is too early to differentiate the hope from the hype; nonetheless, new unforeseen imaging paths have emerged in recent years. From this perspective, the review by C.Romei and colleagues described exhaustively the potential role of CT and MR imaging approaches in MPM diagnosis, staging and assessment of therapy response [6].

Furthermore, it is increasingly evident that the multidisciplinary management of this orphan and rare pathology is appropriate during all the steps of patient management, from diagnosis to simultaneous treatments and to the identification of a truly personalized therapeutic program.
